# Association of NT-proBNP and GDF-15 with markers of a prothrombotic state in patients with atrial fibrillation off anticoagulation

**DOI:** 10.1007/s00392-019-01522-x

**Published:** 2019-07-06

**Authors:** Paweł T. Matusik, Barbara Małecka, Jacek Lelakowski, Anetta Undas

**Affiliations:** 1 Department of Electrocardiology, The John Paul II Hospital, Kraków, Poland; 2 Institute of Cardiology, Jagiellonian University Medical College, 80 Prądnicka Street, 31-202 Kraków, Poland; 3 Krakow Center for Medical Research and Technology, The John Paul II Hospital, Kraków, Poland

**Keywords:** Atrial fibrillation, NT-proBNP, GDF-15, Cardiac troponin, Fibrinolysis, Thrombin generation

## Abstract

**Abstract:**

We investigated whether growth differentiation factor-15 (GDF-15), also known as macrophage inhibitory cytokine-1 (MIC-1), levels are associated with a prothrombotic state in atrial fibrillation (AF) as compared to N-terminal pro-B-type natriuretic peptide (NT-proBNP) and high-sensitivity cardiac troponin I (cTnI-hs). In 103 patients with AF assessed off anticoagulation (age: 71.0 [65.0–76.0] years; CHA_2_DS_2_-VASc score: 4.6 ± 1.7), we measured endogenous thrombin potential (ETP), plasma fibrin clot permeability (*K*_s_, a measure of clot density) and clot lysis time (CLT) and other hemostatic parameters, along with GDF-15, NT-proBNP, and cTnI-hs. GDF-15 positively correlated with ETP and CLT (*r* = 0.25, *P* = 0.01 and *R* = 0.56, *P* < 0.0001, respectively) but not with *K*_s_, von Willebrand factor, thrombin-activatable fibrinolysis inhibitor, plasminogen, antiplasmin or tissue-type plasminogen activator antigen. NT-proBNP showed a stronger association with ETP (*r* = 0.60, *P* < 0.0001) and a similar correlation with CLT (*R* = 0.53, *P* < 0.0001), while cTnI-hs correlated solely with CLT (*R* = 0.25, *P* = 0.01). After adjustment for clinical and laboratory parameters, GDF-15 was a better independent predictor of CLT (unstandardized coefficient *B* 0.009; 95% confidence interval [CI] 0.006–0.012) than NT-proBNP (*B* 0.007; 95% CI 0.004–0.010, *R* (2) = 0.51; *P* < 0.0001); while among the three biomarkers, only NT-proBNP was an independent predictor of ETP. Elevated GDF-15 and NT-proBNP independently predict impaired fibrin clot lysability, while NT-proBNP is a key predictor of heightened thrombin formation in AF. Our findings suggest that a predictive value of NT-proBNP and GDF-15 in AF could be in part attributed to their association with prothrombotic blood alterations.

**Graphic Abstract:**

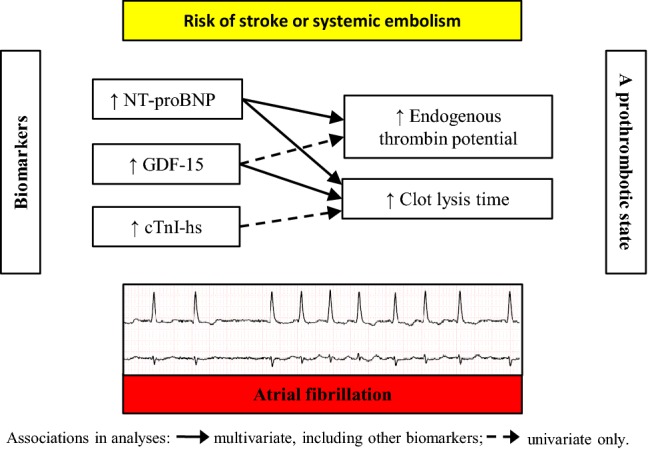

**Electronic supplementary material:**

The online version of this article (10.1007/s00392-019-01522-x) contains supplementary material, which is available to authorized users.

## Introduction

Atrial fibrillation (AF) increases the risk of stroke and systemic thromboembolism. Their current prediction scores are based on clinical variables. Increasing amount of data indicates the potential of cardiac biomarkers to improve prediction of outcomes in patients with AF [1, 2]. Recently, in AF N-terminal pro-B-type natriuretic peptide (NT-proBNP) and high-sensitivity cardiac troponin (cTn-hs) have been shown to be strong and independent predictors of thromboembolism and when combined with clinical data, improve stroke risk assessment [3, 4]. Moreover, the two biomarkers have been reported to be associated with a prothrombotic state in AF [5, 6]. In addition, in patients with AF on anticoagulation, NT-proBNP was not an independent predictor of major bleeding [7, 8].

Growth differentiation factor-15 (GDF-15), also known as macrophage inhibitory cytokine-1 (MIC-1), along with NT-proBNP, cTn-hs, age and heart failure diagnosis, have been reported to predict death in AF [9]. GDF-15 in anticoagulated patients with AF has been found to be strongly associated with increased risk of major bleeding, independently of cardiovascular risk factors and other biomarkers, but only in these complex models, it was not associated with stroke/systemic embolism risk [8, 10–12]. Moreover, there is evidence linking GDF-15 with left atrial/left atrial appendage thrombus presence in AF [13].

Mechanisms linking GDF-15 with thromboembolism in AF are unknown. We investigated the relationship between GDF-15 with prothrombotic abnormalities in AF patients compared with NT-proBNP and high-sensitivity cardiac troponin I (cTnI-hs).

## Methods

In a cross-sectional study, we evaluated patients with AF. We excluded individuals with myocardial infarction or venous thromboembolism within the previous 3 months, kidney failure requiring dialysis or creatinine clearance < 15 mL/min, liver cirrhosis, known cancer and acute infection. Comorbidities and clinical scores were assessed as described [5, 6]. The study protocol was approved by bioethical committee and all patients gave informed consent to participate in the study.

Demographic and clinical data were collected at enrolment. Conventional techniques were used to perform transthoracic echocardiography. Fasting venous blood samples were collected in patients off oral anticoagulation. In patients on oral anticoagulants, who were switched to low-molecular-weight heparin, blood collection was performed after at least 12 h since the last heparin injection. None of the patients received anticoagulation treatment on the day of the study, as reported previously [5]. Apart from routine laboratory investigations, electrochemiluminescence immunoassays (Roche Diagnostics, Mannheim, Germany) were used to measure GDF-15 and NT-proBNP levels. Serum concentrations of cTnI-hs were determined using the ARCHITECT i1000SR (Abbott Laboratories, Abbott Park, IL, USA). Latex immunoassay using a STAR coagulation instrument (Diagnostica Stago, Asnières, France) was used to measure von Willebrand factor (vWF) antigen. Plasma tissue-type plasminogen activator (tPA), plasminogen activator inhibitor-1 (PAI-1) antigen and thrombin-activatable fibrinolysis inhibitor (TAFI) antigen were measured by ELISAs (American Diagnostica, Stamford, Connecticut, USA and Chromogenix, Lexington, Massachusetts, USA). Antiplasmin and plasminogen were determined using chromogenic assays (Diagnostica Stago).

Measurement of endogenous thrombin potential (ETP) was performed using calibrated automated thrombography (CAT; Thrombinoscope BV, Maastricht, the Netherlands) in a 96-well plate fluorometer (Ascent Reader, Thermolat Systems OY, Helsinki, Finland) at 37 °C, as previously described [5, 14, 15]. Briefly, 80 µL of platelet-poor plasma was diluted with 20 µL of tissue factor (TF)-based activator (Diagnostica Stago, Asnières, France) containing 5 pmol L^−1^ recombinant TF, 4 micromolar phosphatidylserine/phosphatidylcholine/phosphatidylethanolamine vesicles and FluCa solution (20 µL; HEPES, pH 7.35, 100 nmol L^−1^ CaCl_2_, 60 mg mL^−1^ bovine albumin and 2.5 mmol L^−1^ Z–Gly–Gly–Arg-amidometylcoumarin). ETP was measured twice [16].

The pore size in fiber networks is indicated by clot permeability. It is proportional to a volume of buffer percolating through a clot under a specific hydrostatic pressure. Assessment of clot permeability was described previously in detail [5, 17]. Briefly, 20 mmol/L calcium chloride and 1 U/mL of human thrombin (Sigma) were added to citrated plasma. Tubes with clots were joined with a reservoir of Tris-buffered saline (0.1 mol/L NaCl, 0.01 mol/L Tris, pH 7.5). The volume flowing for 60 min through the gels was measured. The average size of fiber network pores is reflected by the permeation coefficient (*K*_s_). *K*_s_ was calculated using the following equation:

$$ K_{\text{s}} \, = \,Q\, \times \,L\, \times \,\eta /t\, \times \,A\, \times \,\Delta p, $$

where *Q* is the flow rate in time, *L* is the length of a fibrin gel, *ƞ* is the viscosity of liquid in poise, *t* is percolating time, *A* is the cross-sectional area in cm^2^, Δ*p* is a differential pressure in dyne/cm^2^.

The turbidity method was used to determine plasma clot lysis time (CLT), as described previously [5, 17]. Briefly, citrated plasma was mixed with 15 mmol/L calcium chloride, human tissue factor (10,000-diluted; Innovin, Siemens) at a final concentration of 0.6 pmol/L, phospholipid vesicles (12 µmol/L) and recombinant tPA (60 ng/mL; Boehringer Ingelheim, Ingelheim, Germany). Measurements were performed at 405 nm at 37 °C. The midpoint of the clear-to-maximum-turbid transition indicated clot formation. Therefore, measurement from this time to the midpoint of the maximum-turbid-to-clear transition was defined as CLT.

Continuous variables were expressed as the mean ± standard deviation or median (interquartile range). Normality was assessed using the Shapiro–Wilk test. We stratified patients by median GDF-15 level into two groups, i.e., below (low GDF-15) and above or equal to the median of GDF-15 level (high GDF-15). Continuous variables were compared using Student *t* test or Mann–Whitney *U* test, as appropriate. Correlations were tested using a linear Pearson correlation or Spearman rang test, as appropriate. Categorical variables were described by number (frequency) and compared by Pearson $\chi^{ 2}$ test or Fisher’s exact test (if more than 20% of the cells had an expected count of less than 5 and/or if minimum expected count in the particular table was less than 1) [18]. To assess predictors of ETP and CLT, we performed linear regression analysis. $R^{ 2}$ was calculated, and model adequacy assessment using *F* test was performed. *P* values < 0.05 were considered statistically significant. All statistical analyses were performed with IBM SPSS Statistics (version 24).

## Results

The study group included 103 patients (women, 44.7%; median age 71 [65–76] years) with the mean CHA_2_DS_2_-VASc score of 4.6 ± 1.7 (Table 1). Ninety-nine patients (96.1%) had at least two additional (beyond sex) clinical stroke risk factors. The median GDF-15 was 1661.0 (1094.0–2417.0) pg/mL. GDF-15 correlated with age and body mass index (*R* = 0.25, *P* = 0.01 and *R* = − 0.24, *P* = 0.01, respectively), but not with sex or CHA_2_DS_2_-VASc score. Patients with high GDF-15 (≥ 1661.0 pg/mL) did not differ from the remaining subjects with regard to the type of AF, comorbidities or medications used except for lower prevalence of diabetes and lower use of torasemide in the former group (Table 1). However, patients with diabetes mellitus, those using oral hypoglycemic drug or insulin did not differ from the remainder with regards to GDF-15 level (1491.0 [1075.0–1861.0] vs. 1727.0 [1073.3–2452.5] pg/mL, *P* = 0.30; 1526.0 [1042.0–1867.5] vs. 1705.0 [1094.0–2449.0] pg/mL, *P* = 0.49 and 1415.0 [940.8–2586.5] vs. 1684.0 [1129.3–2425.0] pg/mL, *P* = 0.61, respectively). We observed a trend towards inverse correlation between glomerular filtration rate (GFR) and GDF-15 (*R* = − 0.14, *P* = 0.16). Lower fibrinogen and glucose levels were found in patients with high GDF-15, while high-density lipoprotein cholesterol was higher in this group (Supplementary Table S1). GDF-15 tended to inversely correlate with white blood cell count (*R* = − 0.17; *P* = 0.08) and correlated with NT-proBNP (*R* = 0.27, *P* = 0.006) and cTnI-hs (*R* = 0.28, *P* = 0.004).

**Table 1:** Patient characteristics stratified by median GDF-15 level

Variable	Whole group, *n* = 103	Low GDF-15 (445.3–1628.0 pg/mL, *n* = 51)	High GDF-15 (1661.0–5163.0 pg/mL, *n* = 52)	*P* value
Demographics				
Age (years)	71.0 (65.0–76.0)	69.0 (64.0–75.0)	73.0 (68.0–78.0)	0.01
Male sex, *n* (%)	57 (55.3)	29 (56.9)	28 (53.8)	0.76
BMI (kg/m^2^)	28.4 (25.5–32.4)	30.2 (26.5–33.9)	27.2 (25.0–30.3)	0.004
Type of AF				
Paroxysmal AF, *n* (%)	45 (43.7)	20 (39.2)	25 (48.1)	0.61
Persistent AF, *n* (%)	22 (21.4)	11 (21.6)	11 (21.2)	
Permanent AF, *n* (%)	36 (35.0)	20 (39.2)	16 (30.8)	
AF on the day of blood collection, *n* (%)	70 (68.0)	37 (72.5)	33 (63.5)	0.32
CAD, *n* (%)	53 (51.5)	28 (54.9)	25 (48.1)	0.49
COPD, *n* (%)	11 (10.7)	5 (9.8)	6 (11.5)	0.78
Comorbidities and CVD risk factors				
Hypertension, *n* (%)	85 (82.5)	43 (84.3)	42 (80.8)	0.64
Diabetes mellitus, *n* (%)	41 (39.8)	26 (51.0)	15 (28.8)	0.02
Dyslipidemia, *n* (%)	88 (85.4)	47 (92.2)	41 (78.8)	0.06
Smoking history, *n* (%)	37 (35.9)	15 (29.4)	22 (42.3)	0.17
Previous MI, *n* (%)	25 (24.3)	14 (27.5)	11 (21.2)	0.46
Heart failure/LVD, *n* (%)	75 (72.8)	38 (74.5)	37 (71.2)	0.15
Previous stroke, *n* (%)	14 (13.6)	5 (9.8)	9 (17.3)	0.27
CKD stage 3 or 4, *n* (%)	29 (28.2)	15 (29.4)	14 (26.9)	0.78
CHA_2_DS_2_-VASc score	4.6 ± 1.7	4.5 ± 1.9	4.6 ± 1.6	0.71
HAS-BLED score	2 (1–2)	2.0 (1.0–2.0)	2.0 (1.0–2.0)	0.31
Medications, *n* (%)				
Beta-blocker	83 (80.6)	44 (86.3)	39 (75.0)	0.15
ACE-I	63 (61.2)	32 (62.7)	31 (59.6)	0.75
ARB	16 (15.5)	11 (21.6)	5 (9.6)	0.09
CCB	20 (19.4)	12 (23.5)	8 (15.4)	0.30
Aspirin	32 (31.1)	17 (33.3)	15 (28.8)	0.62
Clopidogrel	5 (4.9)	3 (5.9)	2 (3.8)	0.68*
Statin	75 (72.8)	37 (72.5)	38 (73.1)	0.95
Digoxin	20 (19.4)	6 (11.8)	14 (26.9)	0.05
Amiodarone	13 (12.6)	6 (11.8)	7 (13.5)	0.80
Propafenone	9 (8.7)	6 (11.8)	3 (5.8)	0.32*
Oral hypoglycemic drug	28 (27.2)	17 (33.3)	11 (21.2)	0.17
Insulin	9 (8.7)	6 (11.8)	3 (5.8)	0.32*
Aldosterone antagonist	26 (25.2)	17 (33.3)	9 (17.3)	0.06
Furosemide	21 (20.4)	10 (19.6)	11 (21.2)	0.85
Torasemide	26 (25.2)	18 (35.3)	8 (15.4)	0.02
Hydrochlorothiazide	11 (10.7)	3 (5.9)	8 (15.4)	0.12
Indapamide	17 (16.5)	11 (21.6)	6 (11.5)	0.17
Echocardiographic parameters^a^				
LVEF (%)	47.7 ± 13.2	48.4 ± 14.6	47.0 ± 11.9	0.59
LVEF ≥ 50%, *n* (%)	40 (39.6)	22 (44.0)	18 (35.3)	0.80
LA diameter (cm)	4.6 (4.1–5.0)	4.4 (4.1–5.0)	4.7 (4.1–5.0)	0.33

Data are presented as mean ± standard deviation or median (interquartile range) or number (percentage)

*ACE-I* angiotensin-converting enzyme inhibitor, *AF* atrial fibrillation, *ARB* angiotensin II receptor blocker, *BMI* body mass index, *CAD* coronary artery disease, *CCB* calcium channel blocker, *CKD* chronic kidney disease, *CLT* clot lysis time, *COPD* chronic obstructive pulmonary disease, *CVD* cardiovascular disease, *GDF-15* growth differentiation factor-15, *LA* left atrial, *LVD* left ventricular dysfunction, *LVEF* left ventricular ejection fraction, *MI* myocardial infarction, *n* number

*Fisher’s exact test (exact significance, 2-sided)

^a^The data for LVEF and LA diameter were available for 101 and 95 patients, respectively

Analysis of thrombin generation showed that there is a weak positive association of GDF-15 with ETP (*r* = 0.25, *P* = 0.01; Table 2). ETP strongly correlated with NT-proBNP (*r* = 0.60; *P* < 0.0001) and tended to correlate with cTnI-hs (*r* = 0.19, *P* = 0.05). On linear regression analysis, NT-proBNP (standardized coefficient *β* 0.60; unstandardized coefficient *B* 0.08; 95% confidence interval [CI] 0.06–0.10, $R^{2}$  = 0.36; *P* < 0.0001) and GDF-15 (β 0.25; *B* 0.03; 95% CI 0.008–0.060, $R^{2}$ = 0.06; *P* = 0.01), but not cTnI-hs (*β* 0.19; *B* 11.12; 95% CI − 0.12–22.35, $R^{2}$ = 0.04; *P* = 0.052) predicted ETP. However, when NT-proBNP and GDF-15 were assessed together as predictors of ETP, only NT-proBNP remained a significant predictor (Supplementary Table S2). The same was true after adjustment for age, sex, BMI and fibrinogen (Supplementary Table 3) as well as after additional adjustment for coronary artery disease, heart failure/left ventricular dysfunction, GFR, antiplasmin, PAI-1, TAFI, C-reactive protein (CRP) and cTnI-hs (Table 3).

**Table 2:** Laboratory, coagulation and fibrinolysis parameters stratified by median GDF-15 level

Variable	Whole group, *n *= 103	Low GDF-15 (445.3–1628.0 pg/mL, *n* = 51)	High GDF-15 (1661.0–5163.0 pg/mL, *n* = 52)	*P* value
Laboratory parameters				
GFR (mL/min)	73.0 (56.0–85.0)	74.0 (56.0–85.0)	73.0 (53.0–85.3)	0.70
CRP (mg/L)*	1.7 (1.0–3.4)	1.7 (1.0–3.2)	1.6 (1.0–3.6)	0.73
GDF-15 (pg/mL)	1661.0 (1094.0–2417.0)	1094.0 (834.2–1413.0)	2407.0 (1830.0–3175.8)	< 0.0001
NT-proBNP (pg/mL)	721.0 (401.0–1396.0)	634.0 (440.0–1009.0)	1086.5 (398.8–1694.5)	0.09
cTnI-hs (ng/l)	6.1 (5.0–7.5)	5.7 (4.6–7.1)	6.4 (5.5–8.3)	0.0099
Coagulation and fibrinolysis parameters				
APTT (s)	27.8 ± 3.8	27.5 ± 4.3	28.0 ± 3.2	0.48
INR	1.0 (1.0–1.1)	1.1 (1.0–1.1)	1.0 (1.0–1.1)	0.45
Fibrinogen (g/l)*	3.4 (2.7–4.1)	3.7 (3.2–4.0)	3.2 (2.4–4.1)	0.047
vWF:Ag (%)	187.0 (151.0–234.0)	183.0 (135.0–220.0)	190.0 (155.8–243.8)	0.22
TAFI:Ag (%)	102.0 (94.0–113.0)	105.0 (95.0–113.0)	100.0 (91.0–114.5)	0.51
Plasminogen (%)	105.6 ± 14.3	103.7 ± 12.5	107.4 ± 15.7	0.19
Antiplasmin (%)	106.0 (96.0–115.0)	107.0 (96.0–117.0)	103.5 (95.0–114.0)	0.70
tPA:Ag (ng/mL)	7.2 (5.7–9.8)	7.0 (5.9–8.0)	7.6 (5.5–10.3)	0.49
PAI-1:Ag (ng/mL)	15.6 ± 4.0	15.4 ± 3.8	15.8 ± 4.3	0.67
ETP (nM × min)	1488.0 (1403.0–1578.0)	1453.0 (1374.0–1521.0)	1502.5 (1447.8–1610.3)	0.01
*K*_s_ (× 10^−9^ cm^2^)	6.6 ± 0.9	6.6 ± 0.9	6.6 ± 0.9	0.69
CLT (min)	98.0 (81.0–109.0)	86.0 (76.0–100.0)	102.5 (94.3–117.5)	< 0.0001

Data are presented as mean ± standard deviation or median (interquartile range)

*APTT* activated partial thromboplastin time, *CLT* clot lysis time, *CRP* C-reactive protein, *cTnI-hs* high-sensitivity cardiac troponin I, *ETP* endogenous thrombin potential, *GFR* glomerular filtration rate, *INR* international normalized ratio, *K*_*s*_ clot permeability, *NT-proBNP* N-terminal pro-B-type natriuretic peptide, *PAI-1* plasminogen activator inhibitor-1, *TAFI* thrombin-activatable fibrinolysis inhibitor, *tPA* tissue-type plasminogen activator, *vWF* von Willebrand factor For other abbreviations see Table 1

*The data for fibrinogen and cTnI-hs were available for 101 patients, while the data for CRP were available for 100 patients

**Table 3:** Multiple regression analysis of predictors of ETP and CLT in patients with atrial fibrillation

	Standardized coefficients *β*	Unstandardized coefficients *Β* (95% confidence interval)	*P* value
ETP ($R^{2}$ = 0.40; *P* < 0.0001)*			
NT-proBNP (pg/mL)	0.56	0.07 (0.05–0.10)	<0.0001
GDF-15 (pg/mL)	0.10	0.01 (− 0.01–0.04)	0.29
CLT ($R^{2}$ = 0.51; *P* < 0.0001)*			
NT-proBNP (pg/mL)	0.37	0.007 (0.004–0.010)	<0.0001
GDF-15 (pg/mL)	0.49	0.009 (0.006–0.012)	<0.0001

*Adjusted for age, sex, BMI, CAD, heart failure/LVD, GFR, antiplasmin, PAI-1, TAFI, CRP, cTnI-hs and fibrinogen. For abbreviations see the description of Tables 1 and 2

As expected, *K*_s_ correlated with CLT (*r* = − 0.37, *P* = 0.001) and fibrinogen (*r* = − 0.49; *P* < 0.0001), but was not significantly associated with other cardiovascular biomarkers or fibrinolysis parameters (only trends were observed, data not shown). However, *K*_s_ was inversely correlated with white blood cell count and CRP (for both *r* = − 0.25; *P* = 0.01).

**Fig. 1:**
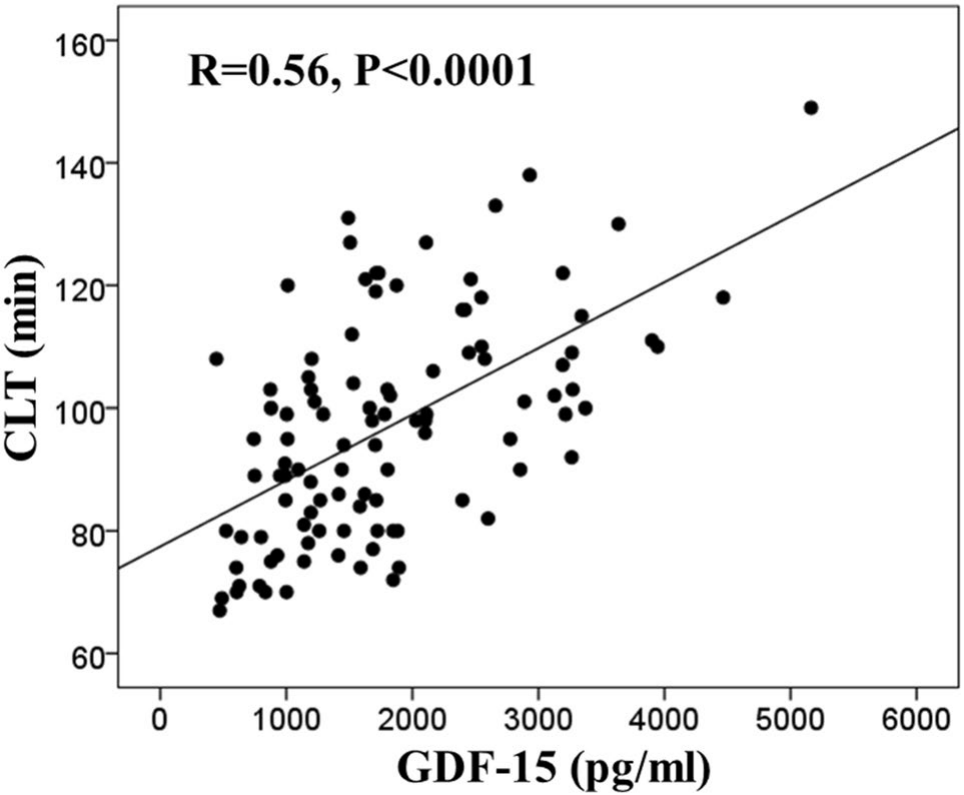
Correlation of clot lysis time (CLT) with growth differentiation factor-15 (GDF-15) in patients with atrial fibrillation

Regarding fibrinolysis, GDF-15 showed no association with TAFI, plasminogen, antiplasmin, tPA or PAI-1 antigen (data not shown). GDF-15 correlated with CLT (*R* = 0.56, *P* < 0.0001; Fig. 1), but not with *K*_s_ (*r* = 0.01, *P* = 0.89). Patients with high GDF-15 had 19.2% longer CLT (Table 2). NT-proBNP correlated with CLT (*R* = 0.53, *P* < 0.0001), and tended to correlate with *K*_s_ (*r* = − 0.17, *P* = 0.10), while cTnI-hs showed weak association with CLT (*R* = 0.25, *P* = 0.01). Moreover, CLT correlated with ETP (*r* = 0.36, *P* = 0.0002). There was a trend towards correlation between CLT and CHA_2_DS_2_-VASc score (*R* = 0.18, *P* = 0.07), without association with age or BMI (data not shown).

CLT was predicted by GDF-15 (*β* 0.57; *B* 0.011; 95% CI 0.008–0.014; $R^{2}$ = 0.33; *P* < 0.0001), NT-proBNP (*β* 0.51; *B* 0.009; 95% CI 0.006–0.012), $R^{2}$ = 0.26; *P* < 0.0001) and cTnI-hs (*β* 0.23; *B* 1.84; 95% CI 0.29–3.39, $R^{2}$ = 0.05; *P* = 0.02). In a model incorporating these three biomarkers, both GDF-15 and NT-proBNP, but not cTnI-hs, predicted CLT (data not shown). GDF-15 and NT-proBNP predicted CLT in unadjusted model (Supplementary Table S2), after adjustment for age, sex, BMI and fibrinogen (Supplementary Table 3) as well as after additional adjustment for coronary artery disease, heart failure/left ventricular dysfunction, GFR, antiplasmin, PAI-1, TAFI, CRP, cTnI-hs (Table 3).

In our cohort vWF, a marker of endothelial dysfunction, showed a weak association with CLT (*R* = 0.24; *P* = 0.01), but was not related to the three biomarkers or fibrinolysis parameters (data not shown).

A median of mean heart rate was 75 (70–80) bpm. There was no correlation between *K*_s_ and CLT and mean heart rate (*r* = 0.03, *P* = 0.77 and *R* = − 0.03, *P* = 0.78, respectively). On the day of blood collection, AF was observed in 68.0% of patients (Table 1). There was no difference in *K*_s_ in patients with and without AF on the day of blood collection (6.7 ± 0.9 × 10^−9^ cm^2^ vs. 6.5 ± 0.8 × 10^−9^ cm^2^; *P* = 0.42). However, there was a tendency towards prolonged CLT (99.0 [83.8–111.3] min vs. 91.0 [78.5–104.5] min; *P* = 0.12) and increased ETP (1495.5 [1433.3–1582.0] nM × min vs. 1450.0 [1327.0–1541.5] nM × min; *P* = 0.08) in patients with AF on the day of blood collection, when compared with the remainder.

## Discussion

The present study shows that in nonanticoagulated patients with AF, GDF-15 and NT-proBNP, after adjustment for clinical and laboratory parameters, are the independent predictors of prolonged clot lysis, a measure of plasma fibrinolytic potential. Only NT-proBNP has been observed to independently predict ETP, which suggests that these biomarkers may reflect various aspects of prothrombotic alterations observed in AF. Importantly, this study supports the evidence that elevated biomarkers in AF indicate higher thrombotic risk that cannot be fully explained by age or CHA_2_DS_2_-VASc score. Our findings suggest that a predictive value of the two biomarkers in AF could be in part attributed to their association with prothrombotic blood alterations. However, mechanisms which underlie associations between these biomarkers and thrombotic tendency remain to be established.

We extend our previous observations on associations of NT-proBNP with CLT and ETP [5]. Here, we have shown that associations of NT-proBNP with these surrogate markers of a prothrombotic state are independent of other clinical and laboratory parameters as well as other biomarkers studied (including cTnI-hs and GDF-15). These data might help to explain observations that NT-proBNP is strongly linked to increased risk of stroke/systemic embolism independently of clinical risk factors and cTn-hs [4], but is not independently associated with major bleeding in AF [7, 8].

To our knowledge, there have been no published reports linking GDF-15 levels in circulating blood with specific hemostatic markers in AF, despite the fact that several studies demonstrated that elevated GDF-15 in AF is linked to increased risk of major bleeding, independently of cardiovascular risk factors and other biomarkers [10, 11]. Some investigators reported an increased risk of stroke or systemic embolism at elevated GDF-15 concentrations in AF patients even on oral anticoagulation [7]. However, a predictive role of GDF-15 in terms of stroke/systemic embolism remains still controversial as evidenced in models when other biomarkers were included, namely NT-proBNP and cardiac troponin T in the Randomized Evaluation of Long-Term Anticoagulation Therapy (RE-LY) biomarker substudy and NT-proBNP, cardiac troponin I and cystatin C in the Apixaban for Reduction in Stroke and Other Thromboembolic Events in Atrial Fibrillation (ARISTOTLE) trial substudy [8, 10, 11]. In these seminal trials, when multivariable analyses including other biomarkers were performed, GDF-15 was no longer independently associated with stroke/systemic embolism [8, 10, 11]. Expression of GDF-15 is induced by pro-inflammatory cytokines, including tumor necrosis factor (TNF)-α, interleukin (IL)-1β and IL-6 [19, 20]. GDF-15 exerts anti-inflammatory effects, leading to inhibition of lipopolysaccharide-stimulated secretion of TNF-α by macrophages [19]. Moreover, GDF-15 has been shown to inhibit the activation of β_1_ and β_2_-integrins on platelets and prevent thrombus formation in mice, with no effect on platelet P-selectin expression and dense granule secretion after stimulation [21]. We observed that GDF-15 tended to inversely correlate with white blood cell count, but we did not find any association of GDF-15 with CRP with even lower fibrinogen at high GDF-15 concentrations, indicating the involvement of proinflammatory mechanisms independent of IL-6. We did not observe relations between GDF-15 and vWF, tPA, or PAI-1 antigen in our patients with AF. However, in a cohort of elderly patients GDF-15 was found to be weakly or moderately related to vWF, PAI-1 activity and tPA antigen, suggesting that in patients with AF, GDF-15 may reflect other prothrombotic alterations [22].

Our key observation reported here is a positive association between GDF-15 and CLT, indicating that processes leading to elevation of GDF-15 drive antifibrinolytic reactions unrelated to increased PAI-1 or antiplasmin. A weak association between GDF-15 and lysis time observed in univariate analysis has been recently reported in patients after acute coronary syndrome (ACS) included in the PLATelet inhibition and patient Outcomes (PLATO) trial [23]. It might be speculated that oxidative stress and inflammation [5, 24, 25] promote both elevation of GDF-15 and prothrombotic alterations, rendering both GDF-15 and CLT potentially valuable markers both in AF and after ACS.

It has been shown that formation of denser fibrin clots is associated with increased risk of thromboembolism in patients with AF on oral anticoagulation [26, 27]. Given an inverse association between clot permeability and CLT, it is possible that CLT has a similar prognostic value. In the light of the available data, associations between clinical outcomes and the biomarkers tested are complex. It remains to be explored in a large prospective cohort study whether a predictive value of GDF-15 in terms of morbidity and mortality in AF [7, 9, 10, 13] is in part mediated by suppressed plasma fibrinolytic potential. The independent association of NT-proBNP with both increased ETP and prolonged CLT is consistent with clinical findings indicating that NT-proBNP is more potent than GDF-15 predictor of thromboembolic risk in AF [8]. Our results support the evolving use of biomarkers in more individualized patient-oriented thromboembolic risk stratification in patients with AF [8, 28]. It may be hypothesized that these biomarkers may be helpful, not only in diagnostics, but also in prediction of future use or decision-making on introduction of interventional procedures, including cardiac pacing, ablation and/or renal sympathetic denervation [29–37].

Several study limitations should be acknowledged. Since the vast majority of the current patients were at high risk of stroke or systemic thromboembolism, our findings could not be referred to low-risk AF patients or those receiving anticoagulant therapy, known to modify clot properties and decrease thrombin formation [38, 39]. The associations observed do not necessarily mean the cause–effect relationship. Some of the relations tested in the current study, most probably due to relatively low number of patients and confounding factors present (comorbidities and cardiovascular disease risk factors), did not reach statistical significance. Determination of proinflammatory cytokines or oxidative stress markers was beyond the scope of this study. Molecular mechanisms behind the association of elevated GDF-15 with hypofibrinolysis in AF remain to be elucidated.

This study demonstrates that elevated GDF-15 and NT-proBNP independently predict low plasma fibrinolytic potential measured in patients with AF off anticoagulation, while NT-proBNP is a key predictor of heightened thrombin formation in AF. The three key biomarkers measured in AF reflect various specific prothrombotic abnormalities, suggesting that determination of the biomarkers might refine characterization of a hypercoagulable state in AF. This study provides data linking NT-proBNP and GDF-15 with prothrombotic blood alterations, which might support the concept of a value of the biomarkers in the estimation of stroke risk in AF.

## Electronic supplementary material

Below is the link to the electronic supplementary material.

Supplementary material 1 (DOCX 28 kb)
