# Supplementary material for: Association of NT-proBNP and GDF-15 with markers of a prothrombotic state in patients with atrial fibrillation off anticoagulation
Source: Clin Res Cardiol. 2019 Jul 6;109(4):426–34. doi: 10.1007/s00392-019-01522-x (PMC7098929; doi:10.1007/s00392-019-01522-x)
Supplement: Supplementary file 1 — Supplementary material 1 (DOCX 28 kb) [file 392_2019_1522_MOESM1_ESM.docx]

**SUPPLEMENTARY MATERIAL**

***Clinical Research in Cardiology***

**Association of NT-proBNP and GDF-15 with markers of a prothrombotic state
in patients with atrial fibrillation off anticoagulation**

**Running head: NT-proBNP, GDF-15 and a prothrombotic state in AF**

**Paweł T. Matusik, MD, PhD, FEHRA^1,2^, Barbara Małecka, MD, PhD^1,2^,**

**Jacek Lelakowski, MD, PhD^1,2^, Anetta Undas, MD, PhD^2,3^**

^1^Department of Electrocardiology, The John Paul II Hospital, Kraków, Poland; ^2^Institute of Cardiology, Jagiellonian University Medical College, Kraków, Poland; ^3^Krakow Center for Medical Research and Technology, The John Paul II Hospital, Kraków, Poland

**Correspondence to:**

Prof. Anetta Undas, M.D., Ph.D.,

Institute of Cardiology, Jagiellonian University Medical College,

80 Prądnicka Street, 31-202 Kraków, Poland.

Phone: +48 12 614 30 04; Fax: +48 12 614 21 20; E-mail: [mmundas@cyf-kr.edu.pl](mailto:mmundas@cyf-kr.edu.pl)

**Supplementary Table S1** Additional laboratory parameters stratified by median GDF-15 level

| Variable | Whole group,  n=103 |  | High GDF-15 (1661.0-5163.0 pg/ml, n=52) | *P* value |
| --- | --- | --- | --- | --- |
|  |  | Low GDF-15 (445.3-1628.0 pg/ml, n=51) |  |  |
| Laboratory parameters | | | | |
| WBC (x1000/µl) | 6.3 (5.2-7.3) | 6.4 (5.6-8.0) | 6.1 (5.1-7.1) | 0.06 |
| Hb (g/dl) | 13.7 (13.0-14.9) | 13.7 (13.0-14.9) | 13.9 (13.0-14.9) | 0.90 |
| Platelets (x1000/µl) | 200.0 (166.0-234.0) | 207.0 (180.0-245.0) | 199.0 (161.3-221.3) | 0.16 |
| Glucose (mmol/l) | 5.8 (5.3-6.4) | 6.0 (5.4-6.8) | 5.7 (5.2-6.1) | 0.03 |
| TC (mmol/l) | 4.0 (3.5-5.0) | 4.0 (3.5-4.6) | 4.1 (3.6-5.1) | 0.41 |
| LDL-C (mmol/l) | 2.3 (1.9-2.8) | 2.3 (1.9-2.9) | 2.3 (1.9-2.8) | 0.73 |
| HDL-C (mmol/l) | 1.3 (1.1-1.6) | 1.2 (1.0-1.6) | 1.4 (1.2-1.7) | 0.045 |
| Triglyceride (mmol/l) | 1.2 (0.9-1.6) | 1.3 (0.9-1.7) | 1.1 (0.9-1.5) | 0.37 |

Data are presented as mean ± standard deviation or median (interquartile range)

*GDF-15* growth differentiation factor-15, *HDL-C* high-density lipoprotein cholesterol, *Hb* hemoglobin concentration, *LDL-C* low-density lipoprotein cholesterol, *n* number, *TC* total cholesterol, *WBC* white blood cell

**Supplementary Table S2** Multiple linear regression analysis of predictors of ETP and CLT in patients with atrial fibrillation (without adjustment for other clinical factors or laboratory parameters)

|  | Standardized coefficients β | Unstandardized coefficients  Β (95% confidence interval) | *P* value |
| --- | --- | --- | --- |
| ETP (R^2^=0.37; P<0.0001) | | | |
| NT-proBNP (pg/ml) | 0.57 | 0.08 (0.05-0.10) | <0.0001 |
| GDF-15 (pg/ml) | 0.10 | 0.01 (-0.01-0.04) | 0.23 |
| CLT (R^2^=0.46; P<0.0001) | | | |
| NT-proBNP (pg/ml) | 0.38 | 0.007 (0.004-0.010) | <0.0001 |
| GDF-15 (pg/ml) | 0.47 | 0.009 (0.006-0.012) | <0.0001 |

*CLT* clot lysis time, *ETP* endogenous thrombin potential, *NT-proBNP* N-terminal pro-B-type natriuretic peptide For other abbreviations see Supplementary Table S1

**Supplementary Table S3** Multiple regression analysis of predictors of ETP and CLT in patients with atrial fibrillation (after adjustment for age, sex, body mass index and fibrinogen)

|  | Standardized coefficients β | Unstandardized coefficients  Β (95% confidence interval) | *P* value |
| --- | --- | --- | --- |
| ETP (R^2^=0.38; P<0.0001) | | | |
| NT-proBNP (pg/ml) | 0.54 | 0.07 (0.05-0.10) | <0.0001 |
| GDF-15 (pg/ml) | 0.11 | 0.02 (-0.01-0.04) | 0.22 |
| CLT (R^2^=0.49; P<0.0001) | | | |
| NT-proBNP (pg/ml) | 0.36 | 0.007 (0.004-0.009) | <0.0001 |
| GDF-15 (pg/ml) | 0.50 | 0.009 (0.006-0.012) | <0.0001 |

For abbreviations see Supplementary Tables S1 and S2
